# Perinatal Psychopathology and Bonding: Implications for Maternal-Infant Attachment and Infant Development

**DOI:** 10.1192/j.eurpsy.2025.1826

**Published:** 2025-08-26

**Authors:** C. M. Grego, C. Araújo, A. Tarelho

**Affiliations:** 1 Psychiatry and Mental Health Department, Local Health Unit of Aveiro, Aveiro, Portugal

## Abstract

**Introduction:**

Perinatal psychopathology, encompassing a spectrum of psychiatric disorders such as perinatal depression, generalized anxiety disorder, post-stress traumatic disorder (PTSD), bipolar disorder, and postpartum psychosis, has been increasingly recognized for its potential to disrupt early mother-infant interactions. These disruptions may have profound consequences on the formation of secure attachment, which is crucial for healthy infant neurodevelopment. Attachment theory posits that early relational experiences shape the foundation of emotional regulation and social functioning, making it essential to understand how maternal psychopathology influences this critical developmental period.

**Objectives:**

This study aims to explore the impact of perinatal psychopathology on the development of attachment between mothers and their infants. Specifically, it seeks to identify how various mental health disorders affect maternal sensitivity and responsiveness, and how these alterations contribute to insecure or disorganized attachment styles in children.

**Methods:**

A comprehensive review of the literature was conducted by searching major medical databases (e.g. PubMed and Google Scholar), giving preference to studies published between 2000 and 2024. Articles were selected based on relevance to perinatal psychopathology and attachment theory. This narrative review includes both quantitative and qualitative studies, clinical trials, and observational research that examine how maternal mental health conditions during pregnancy and the postpartum period affect attachment processes. Key themes and findings were extracted to provide a holistic view of the current state of knowledge on this subject.

**Results:**

The findings align with existing research on the impact of maternal mental health on early attachment, reinforcing the role of maternal sensitivity as a mediator between psychopathology and attachment security. Psychiatric conditions impair the mother’s ability to engage in synchronous interactions with her infant, disrupting the child’s ability to develop a secure base. Moreover, the presence of comorbid psychiatric conditions and chronicity of symptoms were identified as risk factors for more severe attachment disruptions. Pharmacological treatment and psychotherapy were shown to mitigate these effects, highlighting the need for integrated perinatal mental health services.

**Conclusions:**

Perinatal depression, PTSD, and psychosis significantly compromise maternal-infant attachment, increasing the risk of insecure or disorganized attachment styles. Timely psychiatric interventions can enhance maternal mental health and promote healthier attachment outcomes. This review underscores the importance of integrating maternal mental health treatment into perinatal care to prevent long-term developmental issues in children and foster secure attachment relationships.

**Disclosure of Interest:**

None Declared

